# Signatures of differential selection in chloroplast genome between *japonica* and *indica*

**DOI:** 10.1186/s12284-019-0322-x

**Published:** 2019-08-14

**Authors:** Lin Cheng, Jungrye Nam, Sang-Ho Chu, Phitaktansakul Rungnapa, Myeong-hyeon Min, Yuan Cao, Ji-min Yoo, Jee-Su Kang, Kyu-Won Kim, Yong-Jin Park

**Affiliations:** 1 Department of Plant Resources, College of Industrial Science, Kongju National University, Yesan, 32439 Republic of Korea; 2 Center for Crop Breeding on Omics and Artifical Intelligence, Kongju National University, Yesan, 32439 Republic of Korea

**Keywords:** Chloroplast genome, *Indica*, *Japonica*, Domestication, Diversity, Rice

## Abstract

**Background:**

The domestication process of Asian rice (*Oryza sativa* L.) is complicated. It’s well established that *Oryza rufipogon* is the ancestor of Asian rice, although the number of domestication events still controversial. Recently, numerous types of studies based on rice nuclear genome have been conducted, but the results are quite different. Chloroplasts (cp) are also part of the rice genome and have a conserved cyclic structure that is valuable for plant genetics and evolutionary studies. Therefore, we conducted chloroplast-based studies, aiming to provide more evidence for the domestication of Asian rice.

**Results:**

A total of 1389 variants were detected from the chloroplast genomes of 412 accessions obtained through the world. *Oryza sativa* L. ssp. *japonica* exhibited slightly less diversity (*π*) than *Oryza sativa L. indica* and wild rice. The fixation index values (*F*_ST_) revealed that *indica* and *japonica* exhibited farther genetic distances compared with wild rice. Across cp genome, Tajima’s *D* test demonstrated that different selection sites occurred in Asian rice. Principal component analyses (PCA) and multidimensional scaling (MDS) clearly classify the Asian rice into different groups. Furthermore, introgression patterns identified that *indica* and *japonica* shared no introgression events in cp level, and phylogenetic studies showed cultivated rice were well separated from different type of wild rice.

**Conclusions:**

Here, we focus on the domestication of Asian rice (*indica* and *japonica*). Diversity and phylogenetic analyses revealed some selection characteristics in the chloroplast genome that potentially occurred in different Asian rice during the domestication. The results shown that Asian rice had been domesticated at least twice. In additional, *japonica* may experience a strong positive selection or bottleneck event during the domestication.

**Electronic supplementary material:**

The online version of this article (10.1186/s12284-019-0322-x) contains supplementary material, which is available to authorized users.

## Background

Rice is a domesticated specie that adapts to its natural and cultural environment and is a lineage developed by farmers through artificial selection during long-term domestication. For the evolutionary history in African rice, scientists have basically reached a consensus that the African rice (*Oryza glaberrima*) was independently domesticated from the wild progenitor *Oryza barthii* along the Niger river. However, for Asian rice, we have limited knowledge of rice domestication compared to other major crops such as wheat and maize (Brenchley et al. 2012; Matsuoka et al. 2002), which is not commensurate with the status of the world’s major crops. Generally, the evolutionary studies of Asian rice are mainly focused on two species, *indica* and *japonica*. *Indica* is mainly called lowland rice and grows throughout tropical Asia. *japonica* rice typically occurs in temperate East Asia, highlands in Southeast Asia and high altitudes in South Asia (Xiong et al. 2011). Despite both *indica* and *japonica* are wildly explored in phenotypic and genetic, the domestication of Asian rice involved in once or multiple events has a long-term controversy (Londo et al. 2006; Huang et al. 2012)

For domestication studies of Asian rice, there are mainly divided into two groups, one group supports *indica* and *japonica* have single domestication, one supports independent domestication in rice. Single domestication studies post that some domesticated-loci of *japonica* and *indica* are almost the same (Gao and Innan 2008; He et al. 2011). What’s more, demographic modeling of large SNPs data shown that Asian rice was single origins, and first domestication from the wild rice *O. rufipogon* in China between 8200 and 13,500 years ago and then spread to South and Southeast Asia (Molina et al. 2011). Also a study using 1083 cultivars of *O. sativa* and 446 wild rice (*O. rufipogon*) to detect quantitative trait loci of domesticated traits revealed that Asian rice often clustered together at domestication sites, which support single origins of Asian rice (Huang et al. 2012). Recently, multiple domestication events were demonstrated by analyzing the *japonica* gene pool from southern China and the Yangtze valley and *indica* gene pools from Indochina and the Brahmaputra valley (Civáň et al. 2015). Moreover, genetic evidence also revealed multiple independent domestication of Asian rice through complex introgression events by 3 K genome data (Wang et al. 2018). Besides that, the single origin contradicts with the domestication sites observed by introgression events, thus the moderate hypothesis has proposed that some domestication-related genes first appeared in *O. sativa* and then transferred between subspecies through introgressive hybridization (Kovach et al. 2007; Sang and Ge 2007) or multiple origin but single domestication led to domesticated Asian rice (Choi and Purugganan 2018). As described above, there is still widespread controversy about the domestication process of cultivated rice in Asia. Ancient rice varieties that are no longer widely cultivated can provide high-yield strains to feed almost 9 billion of humans. Therefore, exploring the genetic information of *O. sativa* is very important to provide more evidence for the domestication of rice and take lots of important insights into the breeding of elite varieties for sustainable agriculture.

As an important plastid, the chloroplast plays an essential role with highly conserved genome in plant cells. Since the first chloroplast genome sequence (*Nicotiana tabacum*) was submitted (Hiratsuka et al. 1989), more than 1962 complete eukaryotic chloroplast genomes have been sequenced and deposited in National Center for Biotechnology Information (NCBI, https://www.ncbi.nlm.nih.gov). The rice genome includes nuclear genome, chloroplast genome, and mitochondrial (mt) genome, play different roles in rice growth and development. For rice genetics and evolutionary studies, scientists mostly focus on the nuclear genome and pay few attentions to the mt and cp genomes. Recently, many evolutionary studies have been conducted based on mt genomes and cp genomes which provide more insights in genetic studies (Daniell et al. 2016; Gray 2015). However, most of these studies are about mt genome in humans, only a few studies based on rice cp genomes (Shinde et al. 2018; Tao et al. 2014). Chloroplasts contain both highly conserved genes fundamental to plant life and maternally inherited genome, which encoding many chloroplast-specific components (Zhang et al. 2017). Chloroplast is also a representative of organelles in plants compared to animals. Therefore, as the plant-specific organelle, the high conserved and maternal inheritance genome, it’s meaningful to investigate rice evolution and genetic based on the cp genome (Tong et al. 2016).

In this study, we provide evidence for the domestication of *Oryza sativa* based on chloroplast analyses. Firstly, we obtained 358 Asian rice and 54 wild rice from the world to detect the genetic differentiation of rice varieties. The chloroplast genome of *Oryza Nipponbare* [Gene Bank: NC_001320] was chosen as a reference for variant calling among whole genomes. Then, we used comprehensive statistical method to fully infer the evolutionary history of Asian rice. Finally, the bayesian inference tree was constructed to fully explore the domestication of Asian rice. This report focuses on the architecture and genetic of the cp genome, which provides more evidence for the domestication of *Oryza sativa*.

## Results

### Samples distribution

**Fig. 1:**
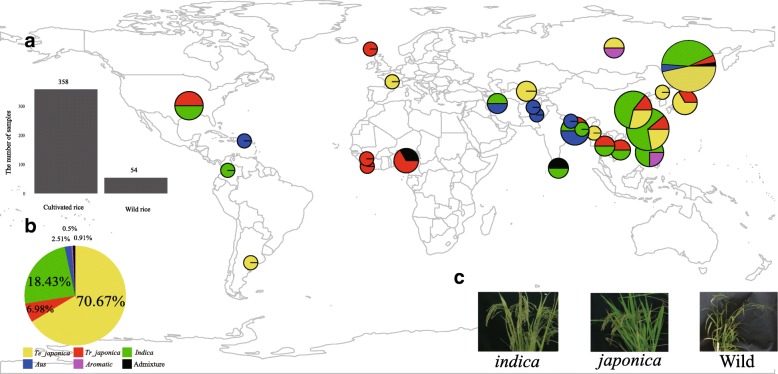
Distribution of 412 rice varieties from the world. (**a**) The total 412 rice varieties are collected from the world, including 358 cultivated varieties and 54 wild varieties. (**b**) The large pie chart summarizes the distribution of subgroups in the 358 Asian rice, and the sample charts on the world map correspond to the country-specific distribution of subgroups (in case of space, some areas have more than one country, but are not display in an intact fashion in this world map). (**c**) The 3 graphs below present some typical phenotypes of *indica, japonica* and wild rice

The genome sequencing and accessions information of all samples are summarized in Additional file 1: Table S1. A total of 475 rice samples were collected from 28 regions of the world’s rice-rich areas and sequenced with a high average coverage (~ 15.88X), yielding ~ 3.42 TB of read data. Since chloroplasts are the maternal inheritance, we removed impure materials based on the previous studies. Finally, 412 purebred rice varieties were chosen from our rice germplasms for their genetic diversity and evolutionary research in chloroplast level (Fig. 1). Of these germplasms, 13.1% were wild rice, and *indica* and *japonica* types occupies 83.5% of whole collection.

A total of 3843 primary variants were detected from 412 samples by using cp reference genomes compared with whole rice genomes. These variants included 2867 SNPs (74.6%) and 976 InDels (25.4%) (Table 1 and Additional file 2: Table S2). After removing variants with > 20% missing calls and minor allele frequency (MAF) < 0.01, 1389 high-quality (HQ) variants were obtained for the subsequent analysis (Daly et al. 2009; Zhao et al. 2008). Of these HQ variants, most were SNPs (87.8%) with only a few numbers of InDels (12.2%). And 82 HQ variants were noted in Asian rice, whereas 1374 HQ variants were found in wild rice. We also annotated gene region of HQ variants in Asian rice. Among these variants, 22 variants (26.83%) were located in the coding areas and 60 variants (73.17%) were shown in non-coding areas. Among the coding region, some important photosynthetic genes, such as *psbI* and *psdD*, did not exhibit any variants (Additional file 3: Table S3). In the subgroups, *indica* harbors numerous variants which similar to *O. rufipogon* and *O. nivara* (Additional file 12: Figure S1a).

**Table 1:** Summary of the SNPs and InDels in chloroplast genome of 358 Asian rice and 54 wild rice

Groups	Number of Accessions		Primary				High-quality			
			Number of variants			Ts/Tv	Number of variants			Ts/Tv
			SNPs	InDels	Total		SNPs	InDels	Total	
Wild rice		54	2836	966	3802	1.772	1162	212	1374	1.741
Cultivated	*Indica*	66	105	101	206	0.764	43	24	67	1.688
	*Te_Japonica*	253	75	86	161	0.531	17	15	32	1.125
	*Tr_Japonica*	25	61	77	138	0.488	11	9	20	0.833
	*Aus*	9	97	100	197	0.831	44	25	69	1.588
	*Aromatic*	2	64	79	143	0.524	13	9	22	1.167
	Admixture	3	62	75	137	0.512	12	9	21	1.000
Total		412	2867	976	3843	1.767	1172	156	1389	1.751

Notes: Ts/Tv is the proportion of transition/transversion. *Te_Japonica*: *temperate japonica*; *Tr_japonica*: *tropical japonica*. Primary: All chloroplast genome variants in our studies. High-quality: High quality variants in chloroplast. Here, we removed 80% of missing data and minor allele frequency (MAF) < 0.01

**Fig. 2:**
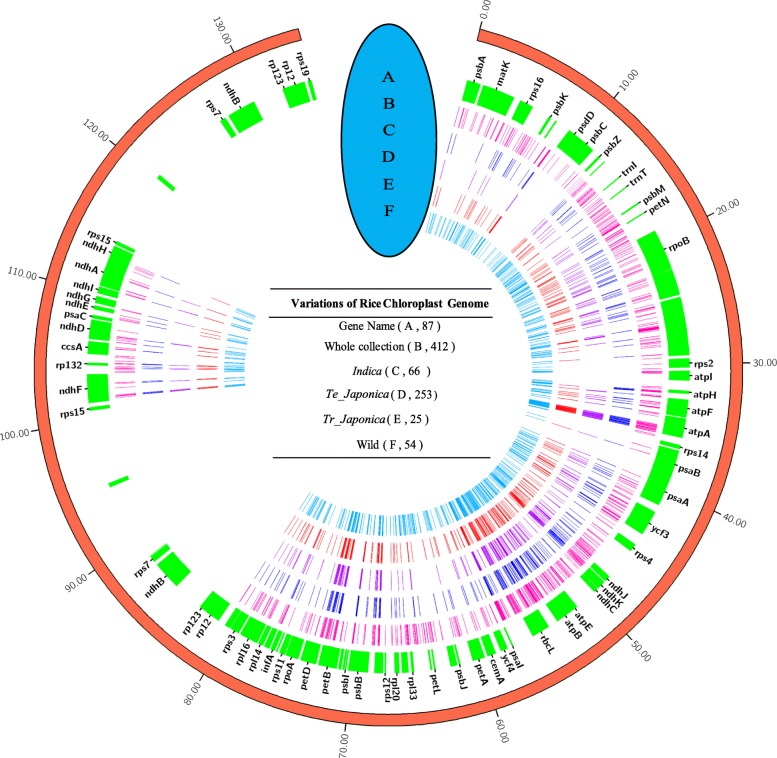
The band distribution of variant (SNPs and InDels) across the chloroplast genome. The band position is depicted as the distance of the first variant of SNPs or InDels based on the reference genome of *Nipponbare*. (**a**-**f**) Highlights marked on the circle map displaying the SNPs and InDels positions. (**a**) The label name of each gene located in the chloroplast genome base on the position of the reference genome. (**b**) Total variants detected among the 412 accessions. (**c**) Variants identified in *indica* subgroup. (**d**) Variants in *temperate japonica* type. (**e**) Variants in the *tropical japonica* type. (**f**) Variants in wild rice. The outside distance unit is kb. The number inside the brackets revealed the number of each accession. In case of the space, not all of the genes were illustrated in the Figure

The overall variants of each groups were summarized, revealing that wild rice had the highest variants both in SNPs and InDels followed by Asian rice *temperate japonica* (75) and *tropical japonica* (70) (Table 1). Since the number of samples in each subpopulation is inconsistent, we have also identified the number of variants per samples in different subgroups (Additional file 2: Table S2). Among the all SNPs, transitions appeared the most frequently, accounting for 63.7%, followed by transversions (36.3%) (Table 1). The variants’ distribution of whole collection and different groups was targeted based on the reference genome, revealing that the whole variants shown cluster distributions (Additional file 2: Table S2). The density of wild rice variants was as high as 10.2/k, and lower density variants were found in Asian rice (0.6/k). In some gene regions, such as *psbA*, the collection of whole samples (Fig. 2b) and wild rice (Fig. 2f) exhibits more variants, while *temperate japonica* shows only a few variants (Fig. 2d).

### Population structure and evolution studies

**Fig. 3:**
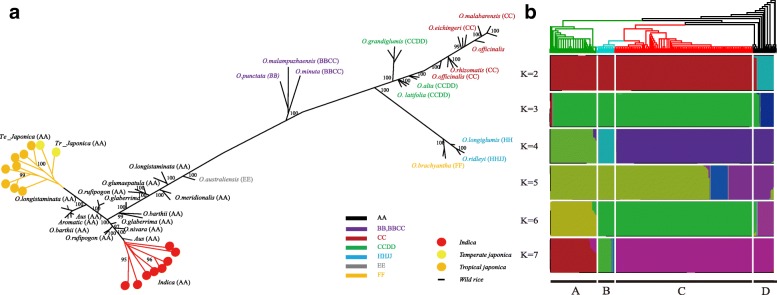
Population structure and the phylogenetic tree of 412 rice accessions. (**a**) The Bayesian Inference tree estimated from MrBayes based on all high-quality SNPs. Here we briefly summarize the characteristics of the tree, the complete tree file we put in Additional file 11. Only the posterior probability bigger than 95 are represents in the figure. (**b**) The combined figure of phylogenetic tree and population structure. Here, the color of the tree is independent of the color of the structure. For population structure, it was conducted by fastStructure using K values from 2 to 7. **a**: *indica* type, **b**: *tropical japonica* type, **c**: *temperate japonica* type, **d**; wild rice

For evolutionary history of Asian rice, phylogenetic tree of bayesian inference (BI) was constructed by MrBayes (Fig. 3a). If *indica* and *japonica* are only domesticated once, a tree with these two subpopulations as sister taxa should be most strongly supported (Yang et al. 2011). However, in our results, all type of *indica* rice was closed to one type of wild rice and whole *japo*nica rice was closed to another type of wild rice (Fig. 3a). In addition, different genomic types also exhibit clustered distributions, the type of rice AA genome exhibit distant genetic distances compared with other genome types. The structure of all samples was conducted using high quality SNPs data to investigate the presence of distinct populations by fastStructure. Assemble populations values from K = 2 to K = 7 were used to entirely distinguish the individual subpopulations among the whole collection. To determine more accurately structure, the K values from 4 to 7 were estimated by ChooseK.py in fastStructure (Fig. 3b). From K = 4 to 7, although candidate K = 5 shown mixture of Asian rice, most of candidates exhibited there were no patterns indicating that *indica* and *japonica* are mixed together, which cannot support single domestication of Asian rice. Briefly, for K = 2 and K = 3, the type of *indica* and *japonica* always mix together and separated from wild rice. For K = 4, we found a division between *indica* and others. It’s indicated the relationship between *japonica* and wild rice was closer than *indica*. For K = 5, *indica* type was well separated from wild rice, where *japonica* type was always mixed with wild rice. From K = 6 to 7, although *indica* and *japonica* type ware separated from each other, there still has a small part of same elements (~ 5%) that occurred in both Asian rice. What’s more, we also found these elements commonly occur in wild rice, which can’t provide evidence for *indica-specific* and *japonica-specific* structure for domestication studies. Because this same structure could be obtained independently from the wild rice during the separate domestication (Civáň and Brown 2018). As described above, the population structure and the phylogenetic studies revealed the separation clusters of Asian rice (*indica* and *japonica*) from wild rice. We also combined with archaeological evidence of Asian rice (more than 9000 years) in China and India (Fuller et al. 2010; Liu et al. 2007), both results suggested that *indica* and *japonica* may have unique background with each other at the cp genome level.

### Haplotype network

**Fig. 4:**
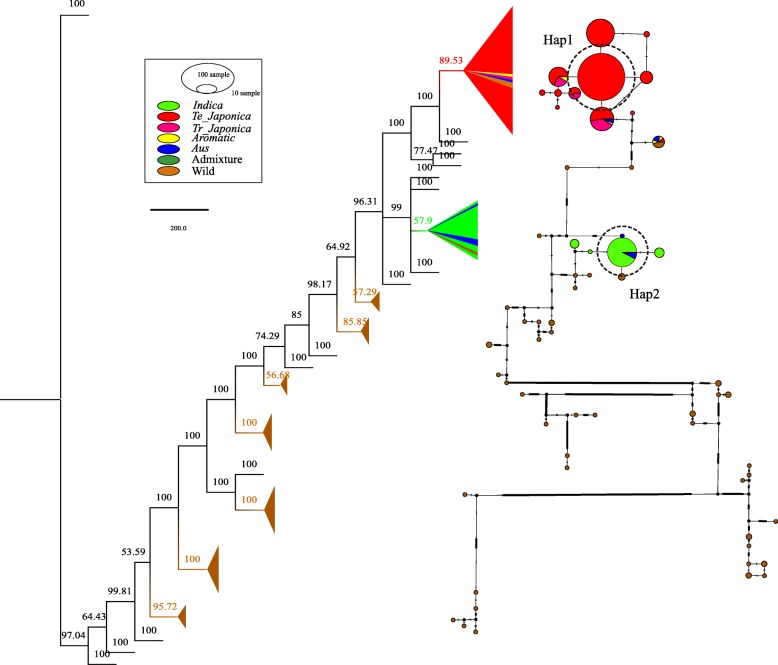
The haplotype tree and TCS network. The haplotype tree and TCS network structure map obtained from 412 samples. The numbers on the tree branches represent confidence values. Different colors represent different populations, and circle size represents the number of samples. Here, haplotypes are mainly dominated by two major haplotypes (dashed circles). Hap1 and Hap2 represent *japonica* type and *indica* type, respectively

In order to find the haplotype relationship between subspecies in rice, we conducted the haplotype analysis based on high quality variants. A total of 60 haplotypes were detected from 412 rice samples. Among these haplotypes, 17 haplotypes and 43 haplotypes were found in Asian rice and wild rice, respectively. In Asian rice, *japonica* exhibited 12 haplotypes, whereas *indica* only exhibited 3 haplotypes. Haplotype results were dominated by two major haplotypes, including primarily the *japonica* type (Hap 1) and *indica* type (Hap 2) (Fig. 4). Hap 1 only covered 144 *japonica* accessions, and Hap 2 harbored 51 *indica* and 4 *aus* accessions (Fig. 4). Previously, an important gene (*Hd1*) which control synchronized flowering in Asian rice, is completely deleted in *O. glaberrima* but this gene is intact in *O. barthii* (the ancestor of *O.glaberrima*), which is suggestive independent domestication (Wang et al. 2014). For our analyses, Asian rice is considered to have been domesticated from wild rice (*O. rufipogon*) thousands years ago (Cheng et al. 2003; Huang et al. 2012). If *indica* and *japonica* have domesticated once, they must have very similar haplotypes. However, we did not identify any share haplotypes between Hap1 and Hap 2. Moreover, this haplotype network was further supported by haplotype tree, which showed that *japonica* and *indica* were well separated from different type of wild rice. As described above, the haplotype analysis supported independent domestication of Asian rice.

### Diversity, introgression and selection analysis

**Fig. 5:**
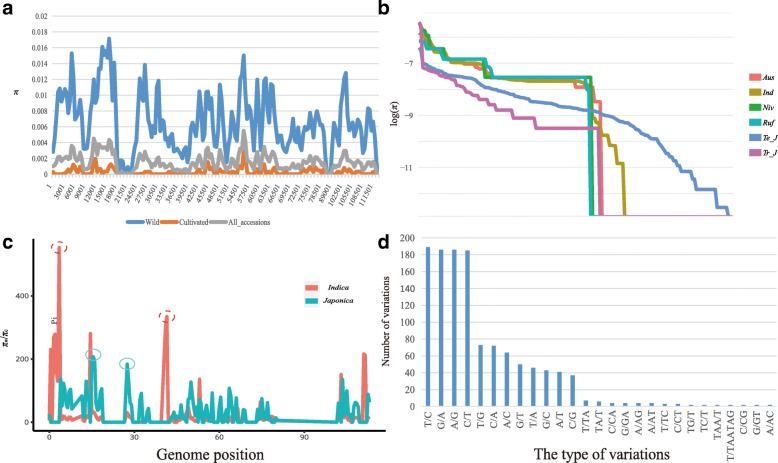
Nucleotide diversity and variant type of all accessions and subgroups. (**a**) Nucleotide diversity of whole collection, Asian rice, and wild rice. The 500 bp window size was used in this analysis. (**b**) Nucleotide diversity of subgroups. The sorted values were plotted in each group. Ind: *indica*; Niv: *O.* nivara; Ruf: *O. rufipogon*; Te_J; *temperate japonica*; Tr_J; *tropical japonica*. (**c**) Reduction of nucleotide diversity (*π*_*w*_/*π*_*c*_) between *indica* and *japonica* in the cp genome. The threshold of top 2.5 percentile is indicated as a red dotted circle for *indica*, blue circle for *japonica*. The regions within the 2.5 percentile are considered as candidate regions under selection. The number of genome position unit is kb. (**d**) Summary of variant types among the cp genome

Nucleotide diversity analysis of the cp genome in the whole accession and different subgroups were conducted based on whole variants (Additional file 4: Table S4). The cp diversity of the whole collection ranges from 5.9 × 10^− 8^ to 5.5 × 10^− 3^. Wild rice exhibited a high nucleotide diversity, whereas Asian rice showed a low level of diversity (Fig. 5a). The diversity of *temperate japonica* and *tropical japonica* is no significant different in cp genome level (*p* value = 0.17). However, the nucleotide diversity of *temperate japonica* was significantly lower than those in *indica* (*p* value < 0.01) (Fig. 5, b and Additional file 5: Table S5). And the mean diversity of *indica* is approximately twice of that of *japonica*. To identify genome regions under selection in Asian rice, we also calculated the nucleotide diversity ratio (*π*_w_/*π*_c_) between *indica* and *japonica* using 1000-bp windows (Fig. 5, c and Additional file 6: Table S6). For each group, 2 regions were identified at the top 2.5 percentile of genetic cutoffs (Wang et al. 2014). These top regions in *indica* group were totally different compared with that regions in *japonica* group. In addition, in some regions of cp genome (30 k–44 k), *japonica* exhibit significant low *π* compared with *indica* (Fig. 5b). This finding may indicate that some areas of *indica* and *japonica* may have been selected during domestication.

To understand the low-diversity of *japonica*, we assessed three hypotheses, including infinite allele mode (IAM), stepwise mutation model (TPM) and two-phased mutation model (SMM) by bottleneck program. These test for recent (within the past 2N_e_ to 4N_e_ generations) population bottlenecks that severely reduce effective population size (N_e_) and produce an excess in heterozygosity to detect the selection effect (http://www1.montpellier.inra.fr/CBGP/software/Bottleneck/pub.html). The results were presented in Additional file 7: Table S7. In the test of IAA model, *indica* showed no excessive heterozygosity (*p* value ≥0.05). However, under the test of three models, we found that both *temperate japonica* and *tropical japonica* exhibited significantly excessive heterozygosity (*p* value < 0.05). Different from *indica*, these analyses suggest that *japonica* may experience a strong positive selection period or bottleneck event.

Introgression events also play an important part in rice domestication (Zhao et al. 2010). To address if there is an introgression event between *indica* and *japonica* in cp genome, we investigated the introgression signal by summarizing statistics based on haplotypes of allele frequency and compared their specific regions (Wang et al. 2018). Generally, the differentiated alleles frequency was identified among *indica* and *japonica*, where the allele is undetected in wild rice. The sites have an allele frequency greater than 0.95 in *japonica* (*japonica-specific*) are compared with that of sites less than 0.05 in *indica*, vice versa. At the all sites, allele information (*indica-specific* type and *japonica-specific* type) of each accession are calling across the chloroplast genome. Although the allele mainly in the range of 0~0.1 and 0.9~1, the results exhibited that *indica-specific* regions were different from *japonica-specific* regions (Additional file 13: Figure S2). It’s indicated there is no introgression event between *indica* and *japonica* in cp genome level (Additional file 8: Table S8). The *Ka*/*Ks* (nonsynonymous substitution rates / synonymous substitution rates) ratio was also calculated to assess the balance among neutral mutations, purifying selection and positive selection (Additional file 12: Figure S1c). This statistical analysis utilized the branch model: a null hypothesis assuming the equational selective pressures across all branches on the phylogenetic tree, and the alternative hypothesis assuming different selective pressures on *indica* and *japonica* as compared with the background branches on the phylogenetic tree. A likelihood ratio test (LRT) was also used to identify accelerated genes in the cp genome. Among all the coding genes, we identified 35 accelerated evolved selection genes (*Ka*/*Ks* > 1) 61 purified selection genes (*Ka*/*Ks* < 1), and one gene (*psbB*) exhibited significantly (*p* value < 0.05) selection on *indica* and *japonica* (Additional file 9: Table S9).

### *F*_ST_, Tajima’s *D* test, PCA and MDS of populations

**Fig. 6:**
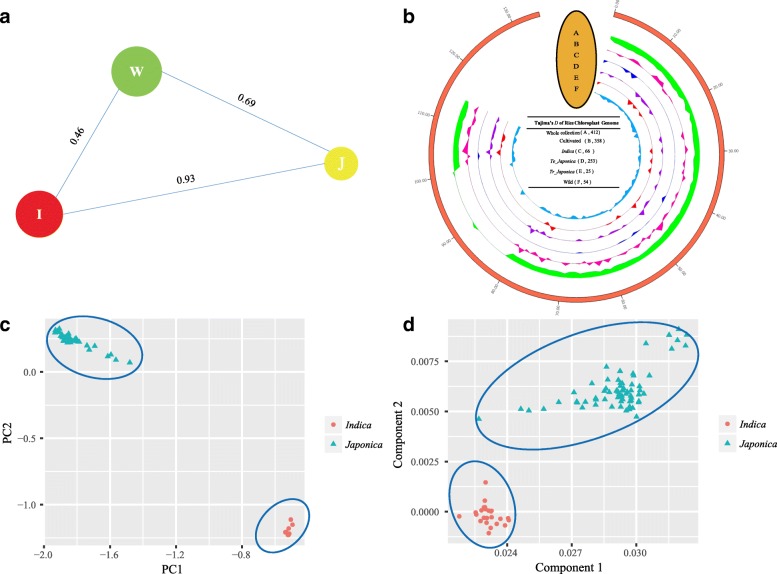
*F*_ST_, Tajima’s *D* test, principal component analysis and multidimensional scaling of populations. (**a**) The *F*_ST_ value between Asian rice and wild rice, the circle size displayed the diversity of each group. The *F*_ST_ value between each group was marked by the length of each line. R: *O. rufipogon*, J: *temperate japonica* and *temperate japonica*. (**b**) Tajima’s *D* values in subgroups. (**c**) Principle component analysis of *indica* and *japonica.* (**d**) Multidimensional scaling plots of *indica* and *japonica*

*F*_ST_ of genetic distances between different populations was calculated based on weighted methods (Weir and Cockerham 1984). We used *F*_ST_ values to determine the degree of differentiation in the subgroups. Comparing with wild rice, *indica* and *japonica* displayed the higher *F*_ST_ value (0.93). This finding indicates a breeding barrier may exist between *indica* and *japonica* and they may be isolated from wild rice for a longer period (Fig. 6a). In domestication studies, one hypothesis supports that *japonica* was first domesticated from *O. rufipogon* and then gradually crossed with wild rice to produce *indica* rice to support the single domestication of rice. This means that *japonica* and *indica* have a close relationship. In our cp *F*_ST_ analysis, these three teams have almost a positive triangular distribution, and *indica* and *japonica* have a relatively far relationship compared to wild rice. The clear separation of *indica* and *japonica* groups of PCA and MDS analyses also confirmed these results (Fig. 6c and d). What’s more, in some cp regions, Tajima’s *D* test showed low values of *indica* whereas much higher values were found in *japonica*, which revealed that Asian rice exhibited the different evolution rates in some specific site (Fig. 6b and Additional file 10: Table S10). As describe above, our findings showed that mainly Asian rice *indica* and *japonica* had far genetic distances, different structural components and unique genetic background, also provided supplemental evidence for nuclear genome research in Asian rice.

## Discussion

### Complementary methods in rice domestication research

Chloroplast-based genome-wide analyses can deep our understanding of plant diversity and genetic information given its highly conserved genome (Liu et al. 2016). The advent of next-generation sequencing (NGS) in the twentieth century has led to rapid advances in the evolutionary analyses (Van Dijk et al. 2014). It is useful to identify genetic and evolution of rice based on the highly conserved cp genome by using NGS data. However, the chloroplast genome only represents the maternal evolutionary history and cannot be fully applied to rapidly differentiate taxa (Tong et al. 2016). Therefore, in some cases, evolutionary studies based on the cp genome should complement the nuclear genome and vice versa. In nuclear genome analyses, studies of same question by different datasets or methods may lead to different results (Huang et al. 2012; Zhao et al. 2018). To acquire more reliable results, nuclear genome evolutionary research should be complemented by cp and mt genome studies.

### Genetic variation and diversity in chloroplast genome

We characterized chloroplast genetic variants in 412 rice samples (Table 1). To obtain reliable data, variants with more than 20% of missing calls and a MAF less than 0.01 were removed from our statistical analyses. Finally, a total of 1389 HQ variants were detected from sequenced data. Although our cp genome analysis involved sequence lengths (~ 134,525) that less than the mt genome length (~ 450,520), our variants values were increased compared with previously values obtained using mt genome (264) (Tong et al. 2017). This may be explained by the fact that the nucleotide substitution rate in mtDNA is less than one-third that in cpDNA (Wolfe et al. 1987). In the 234 high-quality variants of 358 Asian rice, only 0.65 variants per chloroplast genome were noted, which is less than 1% of the total genome. This is consistent with the highly conserved cp genome. 12 types of SNPs (T/C, G/A et al.) and 15 types of InDels (T/TA, C/CA et al.) were identified among high-quality variants (Fig. 5d). Although InDels have many types, the variants are few and some types of InDels like TC/T appeared only 2 times. This finding is reasonable given the highly conserved chloroplast genome because large-scale insertions and deletions are more likely to alter the function of the gene and result in abnormal growth and death. The estimated nucleotide diversity showed that the *π* of *japonica* was lowest compared with *indica* and others (Fig. 5). To figure out this issue, we hypothesized that *japonica* may have experienced a strong positive selection period. After the selection, this species rapidly developed due to the influence of human beings. Therefore, we conducted three model tests (IAM, TPM, and SMM) by bottleneck program. This test confirmed that *japonica* exhibits a significant heterozygosity excess (*p* value < 0.05). Combined with bottleneck events found by 169 nuclear SSRs with two chloroplast loci analyses of *O. sativa* (Garris et al. 2005) and nucleotide genome SNP data (Huang et al. 2012). Our cp analyses indicated that *japonica* experienced a strong selection period or bottleneck event recently.

Some studies using nuclear genome showed low *F*_ST_ values between *japonica* and *indica* which imply frequent gene flows or introgressions (Huang et al. 2012; Huang and Han 2016), and these findings were inconsistent with our result of high *F*_ST_ (0.93), implying a genetic barrier may exist between *japonica* and *indica* and in the cp genome. However, organelle DNA including the cp genome usually exhibits uniparental inheritance, with little or no crossing over, and the rate of evolution is different from that seen in nuclear DNA (McCauley 1995; Whittemore and Schaal 1991). Thus, the inconsistency of the results was due to the different characteristics in inheritance between the genome types. In terms of the recovery of phylogenetic history, it may be advantageous for organelle DNA to be less affected by gene flow that would interfere with the estimation of phylogenetic topology or speciation time.

### The studies of rice domestication

The hypothesis for a single origin of Asian rice is mainly supported by some domesticated traits or gene loci that commonly exist in cultivated rice rather than comparing the genetic differentiation of rice varieties (Molina et al. 2011; He et al. 2011). For multiple domestications, it showed that the different rice subgroups were clustering together and were more closely related to a different type of wild rice based on the phylogenetic analysis (Choi et al. 2017; Wang et al. 2014). Our analysis revealed that the selected regions in *japonica* were different from that of *indica* (Fig. 3). What’s more, the topologies of the phylogenetic tree are consistent with multiple origins of rice (Fig. 5). Specifically, the majority show there is much difference in the selected areas, and the *indica* type is more closely related to one type of wild rice and the *japonica* type is more closely related to another type of wild rice, concordant with previous studies (Londo et al. 2006; Xu et al. 2012). Interestingly, although we mainly focus on the domestication history of *indica* and *japonica*, in our haplotype analysis, we found that the haplotype of *aus* is very similar to *indica*, which is consistent with previous results based on genome SNP data that show *indica* and *aus* have clustered together (Garris et al. 2005; Xu et al. 2012). In addition, we also found that some haplotypes of *aus* accessions may descend from the haplotypes of wild rice and are distinct from the haplotypes of *indica* and *japonica* (Fig. 4). This may indicate that *indica*, *japonica*, and *aus* have independent domestication processes (Liu et al. 2015; Civáň et al. 2015). This paradox may come from the fact that there are some shuttle genes that transfer from the cp genome to the nuclear genome, and the nuclear genome has obtained numerous genes from endosymbiotic organelles during endosymbiotic evolution, which later evolved into the current chloroplasts and mitochondria. In this case, further studies of domestication of *aus* should be the focus on the purest cp genome and exclude the shuttle genes that may come from nuclear and mitochondrial genome.

For evolutionary studies, molecular evidence likes to compare so-called domestication sites such as *sh4* and *prog1* (Li et al. 2006; Tan et al. 2008). These domestication sites are primarily related to phenotype (non-shattering, plant architecture). It has been selected and fixed before becoming the main cultivated rice both in single or multiple domestication events, because of their obvious phenotype (Doebley et al. 2006). Therefore, comparing significant genes associated with phenotype of cultivated subgroups does not make much sense. In order to truly and effectively explore single or multiple domestication events of Asian rice, selection regions but not significantly associated with the phenotype should be focus, such as the chloroplast genome, the mitochondrial genome, and some parts of the nuclear genome. After excluding significant phenotype-related regions, we can compare potential domesticated regions to verify domestication of single or multiple scenarios.

## Conclusions

In this study, 412 rice samples were collected from world rice-rich areas, including 358 Asian rice (*indica*, *temperate japonica*, *tropical japonica*, *aromatic*, *aus* and admixture), and 54 wild rice to investigate the evolution and genetic information of rice. A total of 1389 HQ variants were detected across the cp genome, and the diversity analysis revealed that *indica* has a higher *π* value compared with *temperate japonica* and *tropical japonica*. *F*_ST_ analysis of cp genome shown that *indica* and *japonica* exhibit high *F*_ST_ values. This finding indicated that a breeding barrier may exist between *indica* and *japonica*. Tajima’s *D* test and *Ka*/*Ks* revealed different selection sites occurred between *indica* and *japonica* during the domestication. We also investigated the introgression and selection events in Asian rice*.* The results showed no introgression events between *indica* and *japonica* in cp genome level, but detected a strong signal of recent positive selection in *japonica*. PCA, MDS and population structure showed that *indica* and *japonica* exhibit different compositions and structures. This also confirmed by phylogenetic analysis that *indica* and *japonica* clear separated from different type of wild rice. Moreover, haplotype analysis illustrated no any shared haplotypes between *indica* and *japonica* type. Our analysis uses a variety of methods to demonstrate the independent domestication events of Asian rice. We hope these results provide additional evidence for further rice chloroplast genome genetic and evolutionary studies.

## Methods

### Samples and resequencing

A heuristic set containing 358 rice accessions with 3 types of accessions (landraces, weedy, bred) previously generated from worldwide varieties collected from the National GeneBank of the Rural Development Administration (RDA-Genebank, Republic of Korea) using the program PowerCore (Kim et al. 2007) was selected for whole genome resequencing (Kim et al. 2016). In addition, 54 wild rice accessions were obtained from the International Rice Research Institute (IRRI) in 2017.

For the 358 Asian rice and 54 wild rice accessions from our database, plants are planted in a soft field with enough water. After the heading date (approximately 13 days), young leaves were sampled from a single plant and stored at − 80 °C prior to genomic DNA extraction using the DNeasy Plant Mini Kit (Qiagen). Qualified DNA was used for whole-genome resequencing of the collected rice varieties with an average coverage of approximately 15X on the Illumina HiSeq 2000 Sequencing Systems Platform.

### Variant calling and data management

The assembly process includes data preparation, filtering, mapping, sorting, and variant calling. First, the index is processed by Burrows-Wheeler Alignment v 0.7.15 (BWA) (Li and Durbin 2009), Samtools v1.3.1 (Li et al. 2009) and picard v 2.14 (http://broadinstitute. github. io/picard/) before variant calling. Second, raw data were aligned to the *Nipponbare* cp genome sequence (GenBank: NC_001320) using BWA. A sequence alignment map (SAM) file was created during the mapping and converted to a binary SAM (BAM) file with sorting. Then, removal of duplicates and the addition of reading group IDs were performed using picard Tools. Final realignment and identification of variants were performed using GATK v 3.7. The raw variant call format file (VCF format) of all accessions are available at European Variant Archive (https://www.ebi.ac.uk/eva/?Study-Browser) under the project ID PRJEB28236. Statistical analyses were applied to summarize the number and distribution of variants based on the HapMap (Haplotype Map) file generated from the VCF file. Default settings were used for the most software and tools.

### Statistical analysis and PCA

Statistical analyses of nucleotide diversity (*π*) and population genetic distance (*F*_ST_) were conducted using Vcftools v 0.1.15 (Danecek et al. 2011) with a 1000-bp slide window and 500-bp steps for all collection and individuals. The *F*_ST_ value is used to determine the degree of population differentiation. The significance level of diversity in the group was assessed using t-tests. For introgression events analysis, we followed Zhao’s method (Zhao et al. 2018). The selection effect of the geographic population was generated using Bottleneck v 1.2.02 (Cornuet and Luikart 1996; Piry et al. 1999) according to the allele frequency of each site. Regarding the reliability of the results for the detection of population bottleneck effects, the minor allele frequency < 0.05 are removed from our data. Three mutation models provided by the software are used: infinite allele model (IAM), stepwise mutation model (SMM) and biphasic two-phased mutation model (TPM). To evaluate the relationship and population structure, PCA and MDS were conducted using TASSEL5 based on the high-quality SNPs to provide basic evidence of the population structure. Data were displayed with different groups and colors using R package (ggplot2 (https://cran. r-project. org/web/packages/ggplot2/index. html).

### Haplotype network and Ka/Ks ratios

The TCS (Clement et al. 2000) haplotype network was generated using PopART v 1.7 (Leigh and Bryant 2015). First, we used a python script to make fasta data from variant calling file. Then, fasta data alignment and transformation to nex format was performed using MEGA7 (Kumar et al. 2016). DnaSP v6 (Rozas et al. 2017) was employed for haplotype analysis. Finally, based on the haplotype analysis, *O. officinalis* as the outgroup, the likelihood score tree was produced using the default HKY substitution model and 1000 replications by PAUP4. For *Ka*/*Ks* analyses, all cp orthologous genes from 23 species were aligned to the paml format using prank (Löytynoja 2014). Gblocks v 0.91b (Castresana 2000) was applied to eliminate the conservation area of the ML tree (MEGA7) (Kumar et al. 2016). The branch model, an maximum likelihood method implemented in codeml of PAML v 4.9 h (Yang 2007) was used to estimate the *Ka*/*Ks*, where the ω is a parameter represents the *Ka*/*Ks* with F3X4 codon frequencies. The branch test of the null hypothesis (model = 0) were used for a single background ω across all branches, and test of alternative hypothesis (model = 2) were used for a different ω between the foreground and the background. The likelihood ratio test (LRT) was used to identify accelerated genes in the rice group. Here, *indica* and *japonica* were assigned as foreground branches and others accessions were assigned as background branches. Genes with ω > 5 were removed because they were considered outliers (Castillo-Davis et al. 2004).

### Population structure and evolution research

Briefly, fastStructure v 1.0 (Raj et al. 2014) was used to investigate population clusters. First, InDels were removed from all HQ variants from variant calling file (VCF), and the VCF format was converted into Ped format using Vcftools v 0.1.15. Finally, the Bed format was generated by Plink v1.07 using Ped fime (Purcell et al. 2007). The Bed file was used to estimate different kind of structure in software. Given increased K values ranging from 2 to 7, the subpopulation of an individual ancestry could be completely investigated. Bayesian inference methods were applied to construct a phylogenetic tree for 412 accessions based on the HQ variants. The best substitution model GTR + R + I (general time reversible + gamma distribution + proportion of invariable sites) was detected from 88 models by the software of JModelTest v 2.1.10 (Darriba et al. 2012) using Akaike Information Criterion (Posada and Buckley 2004). The BI tree was constructed by MrBayes v 3.2.5 with a Markov chain Monte Carlo (MCMC) method, convergence with 5.8 × 10^9^ of generation, and 4 chains.

## Additional files

Additional file 1: **Table S1.** Summarize the information of the total samples. (XLSX 51 kb)

Additional file 2: **Table S2.** Summary of the total variant among the subgroups. (XLSX 15 kb)

Additional file 3: **Table S3.** Location of all the SNPs and InDels in their coding region in the reference detected in this study. (XLSX 114 kb)

Additional file 4: **Table S4.** Comparison of nucleotide diversity among all samples, Asian rice and wild rice. (XLSX 17 kb)

Additional file 5: **Table S5.** Comparison of nucleotide diversity of *indica*, *O. rufipogon*, *O. nivara* and *japonica*. (XLSX 18 kb)

Additional file 6: **Table S6** Reduction of nucleotide diversity between indica and japonica in the cp genome. (XLSX 16 kb)

Additional file 7: **Table S7.** IAM, TPM and SMM hypothesis of selection events. (XLSX 10 kb)

Additional file 8: **Table S8.** Allele frequency information of *indica*, *temperate japonica* and *tropical japonica*. (XLSX 23 kb)

Additional file 9: **Table S9** Ka and Ks value among chloroplast genome. (XLSX 17 kb)

Additional file 10: **Table S10.** Comparison of Tajima’s D values of all accession and subgroups. (XLSX 15 kb)

Additional file 11: The result of bayesian inference tree. (TRE 190 kb)

Additional file 12: **Figure S1.** Number of variants in subgroups and Ka/Ks value of all genes in the cp genome. (a) Venn diagram of Asian rice (*temperate japonica*, *tropical japonica*, *aromatic*, *aus* and *indica*). (b) The number of variants in *japonica*, *indica*, *O. rufipogon* and *O. nivara*. The number in the figure indicates same SNP position in each population, and different colors represent different subgroups. (c) The decrease Ka/Ks values of 97 effective selection genes in 23 typical rice accessions. (PDF 1960 kb)

Additional file 13: **Figure S2**. The joint number distribution of allele frequencies in *japonica* and *indica*. (a). The joint number of *indica* and *temperate japonica* based on their allele frequencies. (b). The joint number of *indica* and *tropical japonica* based on their allele frequencies. (c). The frequency of site of *indica* and *temperate japonica* for introgression event. (d) . The frequency of site of *indica* and *tropical japonica* for introgression event. Here, we marked the site (yellow) of frequency bigger than 95% or small than 5%. (PDF 483 kb)

## Data Availability

The datasets supporting the conclusions of this article are included within the article and its additional files. In addition, the raw VCF file generated from current 412 rice accessions was also deposited in the European Variant Archive Database under project ID PRJEB28236, and will be publicly available in Study Browser (https://www.ebi.ac.uk/eva/?Study-Browser).

## Abbreviations

BI: Bayesian inference
Cp: Chloroplast
HQ Variants: High-quality variants, without 80% of missing data and minor allele frequency (MAF) < 0. 01
InDels: Insertion and deletion
MDS: Multidimensional scaling
ML: Maximum likelihood
mt: Mitochondrial
NGS: Next-generation sequencing
NJ: Neighbor-joining
PCA: Principal component analysis
RDA: Rural development administration
SNPs: Single nucleotide polymorphism
