# Supplementary figures and images for: Signatures of differential selection in chloroplast genome between japonica and indica
Source: Rice (N Y). 2019 Aug 14;12:65. doi: 10.1186/s12284-019-0322-x (PMC6692809; doi:10.1186/s12284-019-0322-x)

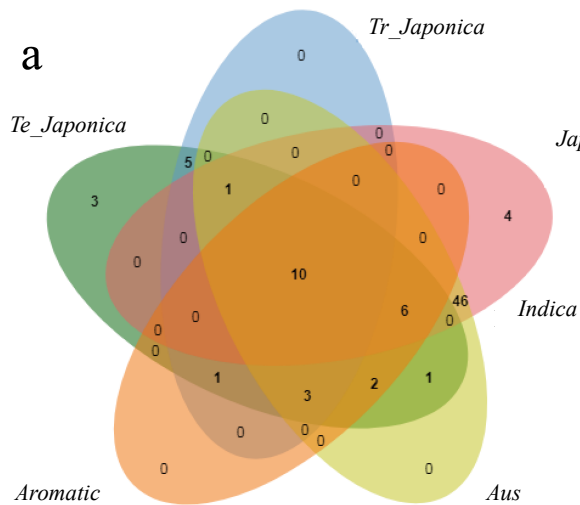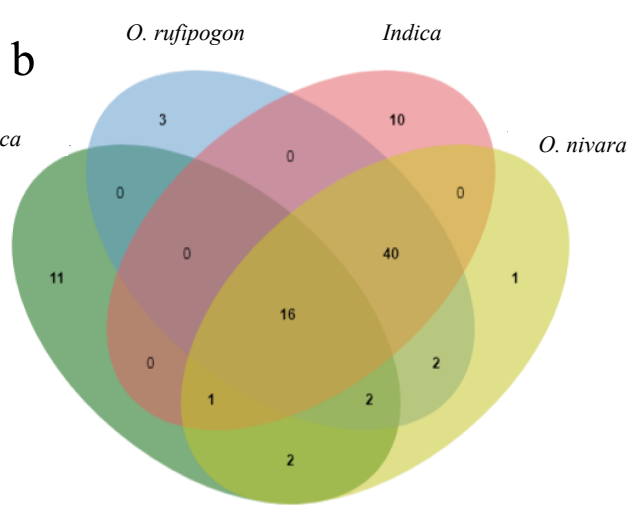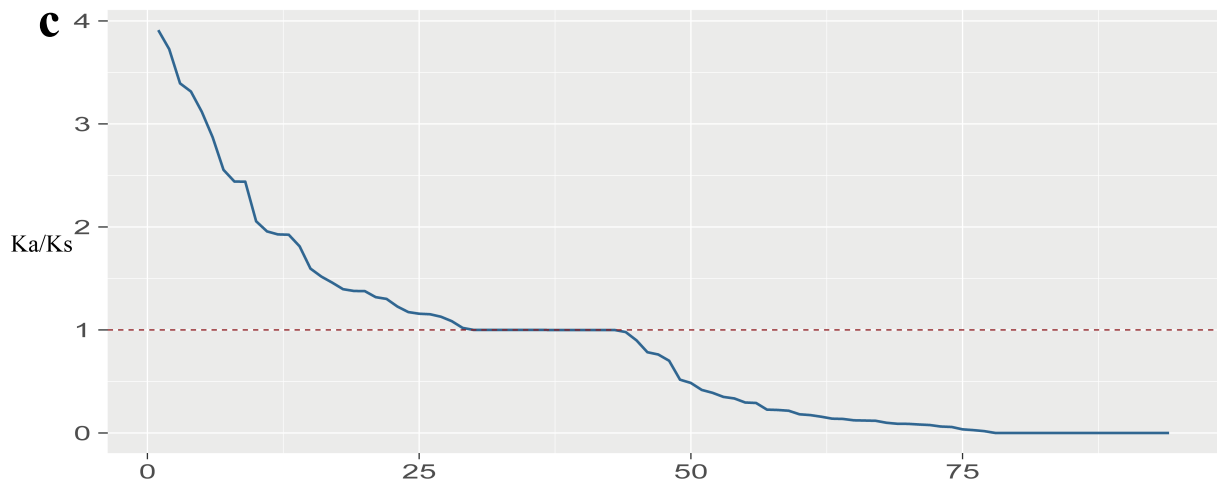

Supplement: Supplementary file 12 — Figure S1. Number of variants in subgroups and Ka/Ks value of all genes in the cp genome. (a) Venn diagram of Asian rice (temperate japonica, tropical japonica, aromatic, aus and indica). (b) The number of variants in japonica, indica, O. rufipogon and O. nivara. The number in the figure indicates same SNP position in each population, and different colors represent different subgroups. (c) The decrease Ka/Ks values of 97 effective selection genes in 23 typical rice accessions. (PDF 1960 kb) [file 12284_2019_322_MOESM12_ESM.pdf]

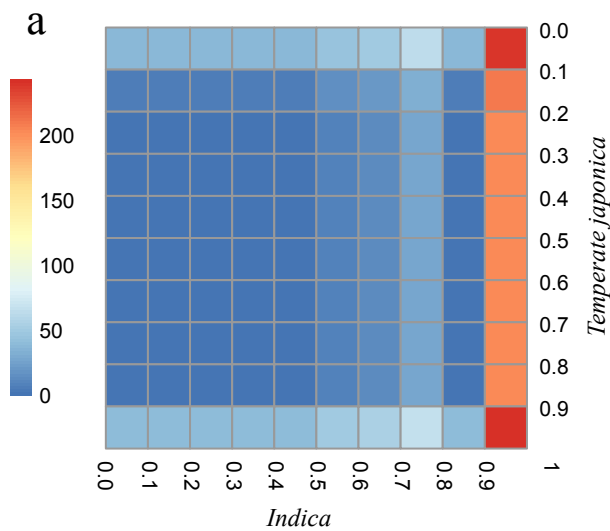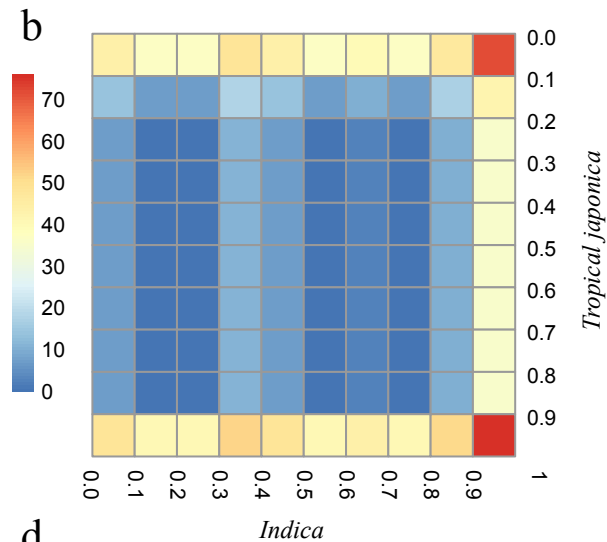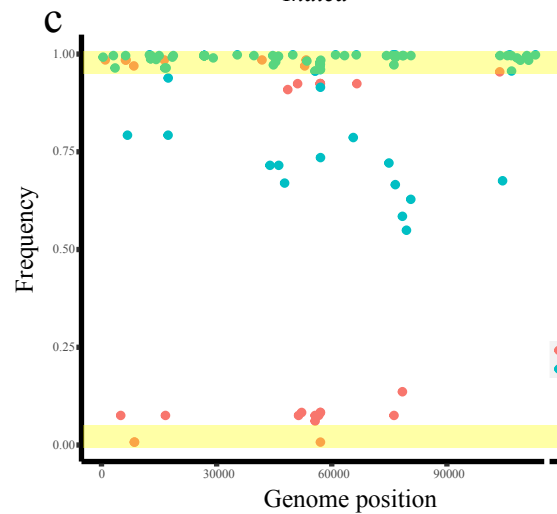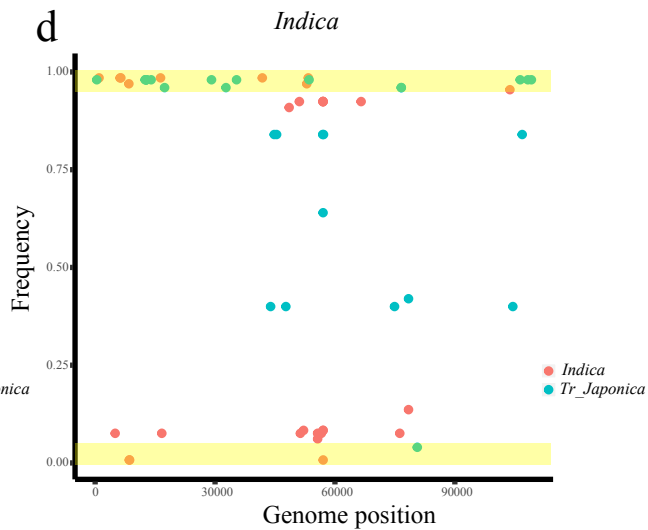

Supplement: Supplementary file 13 — Figure S2. The joint number distribution of allele frequencies in japonica and indica. (a). The joint number of indica and temperate japonica based on their allele frequencies. (b). The joint number of indica and tropical japonica based on their allele frequencies. (c). The frequency of site of indica and temperate japonica for introgression event. (d) . The frequency of site of indica and tropical japonica for introgression event. Here, we marked the site (yellow) of frequency bigger than 95% or small than 5%. (PDF 483 kb) [file 12284_2019_322_MOESM13_ESM.pdf]
